# Cholestatic Jaundice Without Hepatomegaly as the Initial Manifestation of Hepatic Amyloid Light-Chain (AL) Amyloidosis: A Case of Rapid Multiorgan Failure

**DOI:** 10.7759/cureus.110667

**Published:** 2026-06-11

**Authors:** Navanita Biswas, Motaz Almahmood, Shobha Mandal, Jian J Fu

**Affiliations:** 1 Internal Medicine, Tower Health, Reading Hospital, Reading, USA; 2 Internal Medicine, Tower Health Medical Group, Phoenixville, USA; 3 Hematology and Oncology, Tower Health Medical Group, West Reading, USA; 4 Pathology, Tower Health, Reading Hospital, Reading, USA

**Keywords:** al amyloidosis, cholestatic jaundice, hepatic amyloidosis, multiorgan failure, plasma cell neoplasm

## Abstract

Systemic amyloid light-chain (AL) amyloidosis is a rare plasma cell disorder characterized by extracellular deposition of misfolded immunoglobulin light chains, resulting in progressive organ dysfunction. Although hepatic involvement is recognized in AL amyloidosis, it is often clinically silent or presents with hepatomegaly (liver enlargement) and cholestatic liver enzyme abnormalities. Severe cholestatic jaundice as the initial and dominant manifestation is uncommon, particularly in the absence of hepatomegaly or radiographic biliary obstruction.

We report a 63-year-old woman with limited prior healthcare engagement who presented with persistent left ankle pain after a minor twisting injury and was incidentally found to have painless progressive jaundice. Initial laboratory evaluation showed a marked cholestatic pattern of liver injury, including alkaline phosphatase greater than 2300 U/L, total bilirubin of 11.8 mg/dL, direct bilirubin of 8.7 mg/dL, and gamma-glutamyl transferase of 1152 U/L, with disproportionately lower transaminase elevation. Imaging showed cholelithiasis but no biliary obstruction, ductal dilation, focal hepatic lesion, or hepatomegaly. Serologic evaluation for common hepatobiliary causes was unrevealing. Liver biopsy demonstrated amyloidosis predominantly around the portal veins, canalicular and chronic cholestasis, periportal fibrosis, and focal bridging fibrosis. Hematologic evaluation revealed an IgG lambda monoclonal protein, lambda-predominant free light-chain elevation, and bone marrow involvement by a lambda-restricted plasma cell neoplasm, establishing systemic AL amyloidosis with hepatic involvement. Despite initiation of attenuated daratumumab, bortezomib, and dexamethasone therapy, the patient developed rapidly progressive hepatic dysfunction, decompensated heart failure, acute kidney injury, refractory volume overload, and anuric renal failure. She ultimately elected comfort-focused care and was discharged to hospice.

This case highlights hepatic AL amyloidosis as an important diagnostic consideration in unexplained cholestatic jaundice, even without hepatomegaly or biliary obstruction. Early recognition is essential, as severe hepatic dysfunction with multisystem involvement carries a poor prognosis.

## Introduction

Systemic amyloid light-chain (AL) amyloidosis is a plasma cell dyscrasia characterized by extracellular deposition of misfolded immunoglobulin light chains in tissues and organs [1]. These amyloid deposits can disrupt normal organ architecture and function, resulting in a wide spectrum of clinical manifestations depending on the organs involved. Commonly affected organs include the heart, kidneys, liver, gastrointestinal tract, peripheral nerves, and autonomic nervous system [2].

Hepatic involvement in AL amyloidosis is not uncommon histologically, but it is often clinically silent or presents with nonspecific abnormalities [3]. When clinically apparent, hepatic amyloid deposition most commonly manifests with hepatomegaly and elevated alkaline phosphatase. Amyloid deposition within the hepatic sinusoids, portal tracts, and periportal regions may disrupt hepatic architecture and impair bile flow, resulting in intrahepatic cholestasis even in the absence of extrahepatic biliary obstruction. Accumulation of amyloid in the liver has been reported to cause hepatomegaly in 33%-92% of patients, along with variable degrees of cholestasis and jaundice. However, cholestatic jaundice as the primary manifestation of AL amyloidosis is rare and has been reported in fewer than 5% of cases [4,5].

Severe cholestatic jaundice due to hepatic AL amyloidosis may mimic more common hepatobiliary disorders, including biliary obstruction, primary biliary cholangitis, autoimmune liver disease, viral hepatitis, drug-induced liver injury, and infiltrative malignancy. This diagnostic overlap may delay recognition, particularly when imaging does not show biliary obstruction and when classic findings such as hepatomegaly are absent. Early diagnosis is important because AL amyloidosis can progress rapidly, especially when hepatic involvement occurs together with cardiac, renal, or gastrointestinal/autonomic disease. Prompt recognition allows timely initiation of plasma cell-directed therapy and appropriate supportive care; however, outcomes remain poor in patients presenting with advanced hepatic dysfunction and multiorgan involvement.

We report an unusual case of systemic AL amyloidosis presenting initially as severe cholestatic jaundice without radiographic biliary obstruction or hepatomegaly. The patient was subsequently found to have hepatic-dominant AL amyloidosis with rapid progression to cardiac, renal, and multisystem involvement, ultimately resulting in multiorgan failure. This case highlights the importance of considering hepatic amyloidosis in patients with unexplained cholestatic liver injury, even in the absence of hepatomegaly.

## Case presentation

A 63-year-old woman with no significant prior medical history and limited prior healthcare engagement presented to the emergency department in January 2026 with persistent left ankle pain and swelling following a minor twisting injury. During evaluation, she was incidentally noted to have scleral icterus and jaundice, which she reported had been painless and progressively worsening over several weeks. She denied abdominal pain, fever, pruritus, weight loss, alcohol use, or recent medication changes. On presentation, she was hemodynamically stable, with blood pressure of 121/61 mmHg, heart rate of 75 beats/minute, temperature of 36.7°C, respiratory rate of 20 breaths/minute, oxygen saturation of 96% on room air, and weight of 77.7 kg. Physical examination was notable for jaundice and scleral icterus, without hepatomegaly, splenomegaly, macroglossia, or periorbital purpura. Left ankle radiography showed no fracture or dislocation, and her ankle pain was managed conservatively with elevation, topical diclofenac, and cold compresses, with subsequent resolution.

Initial laboratory evaluation revealed a marked cholestatic pattern of liver injury, with alkaline phosphatase greater than 2300 U/L, total bilirubin of 11.8 mg/dL, direct bilirubin of 8.7 mg/dL, and gamma-glutamyl transferase of 1152 U/L. Transaminases were moderately elevated, with aspartate aminotransferase of 318 U/L and alanine aminotransferase of 137 U/L. Additional laboratory testing showed total protein of 8.3 g/dL, hypoalbuminemia with albumin of 1.9 g/dL, leukocytosis with white blood cell count of 17.3 × 10⁹/L, hemoglobin of 11.6 g/dL, and thrombocytosis with platelet count of 529 × 10⁹/L. The calculated globulin gap was approximately 6.4 g/dL, which provided an additional early clue to an underlying monoclonal gammopathy or plasma cell disorder. Coagulation studies showed a prothrombin time of 14.4 seconds and an international normalized ratio of 1.1. An extensive serologic evaluation, including anti-smooth muscle antibody, antimitochondrial antibody, tissue transglutaminase IgA, herpes simplex virus testing, and viral hepatitis panel, was negative. 

Computed tomography of the abdomen demonstrated cholelithiasis without evidence of biliary obstruction. Magnetic resonance imaging of the abdomen showed early morphologic changes suggestive of cirrhosis but no focal hepatic lesions or biliary dilation. Duplex ultrasonography of the lower extremities was negative for deep vein thrombosis. Given persistent unexplained cholestasis, hepatology recommended image-guided liver biopsy, and the patient was empirically started on ursodiol.

**Figure 1:**
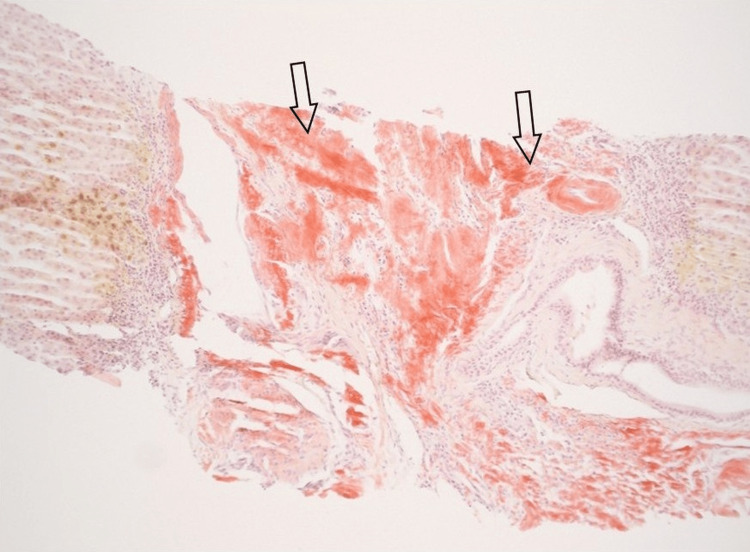
Congo red-positive hepatic amyloid deposition Liver biopsy (Congo red, ×10) showing salmon-pink amyloid deposits (arrows) in the portal and periportal regions, with adjacent cholestatic hepatocytes.

Histopathologic evaluation of the liver biopsy demonstrated amyloidosis, with amyloid deposition predominantly around the portal veins. The biopsy also showed canalicular and chronic cholestasis, periportal fibrosis, and focal bridging fibrosis, supporting hepatic amyloid involvement as the likely cause of the patient’s severe cholestatic presentation. Congo red staining performed on paraffin sections showed Congo red-positive tissue deposits (Figure 1).

**Figure 2:**
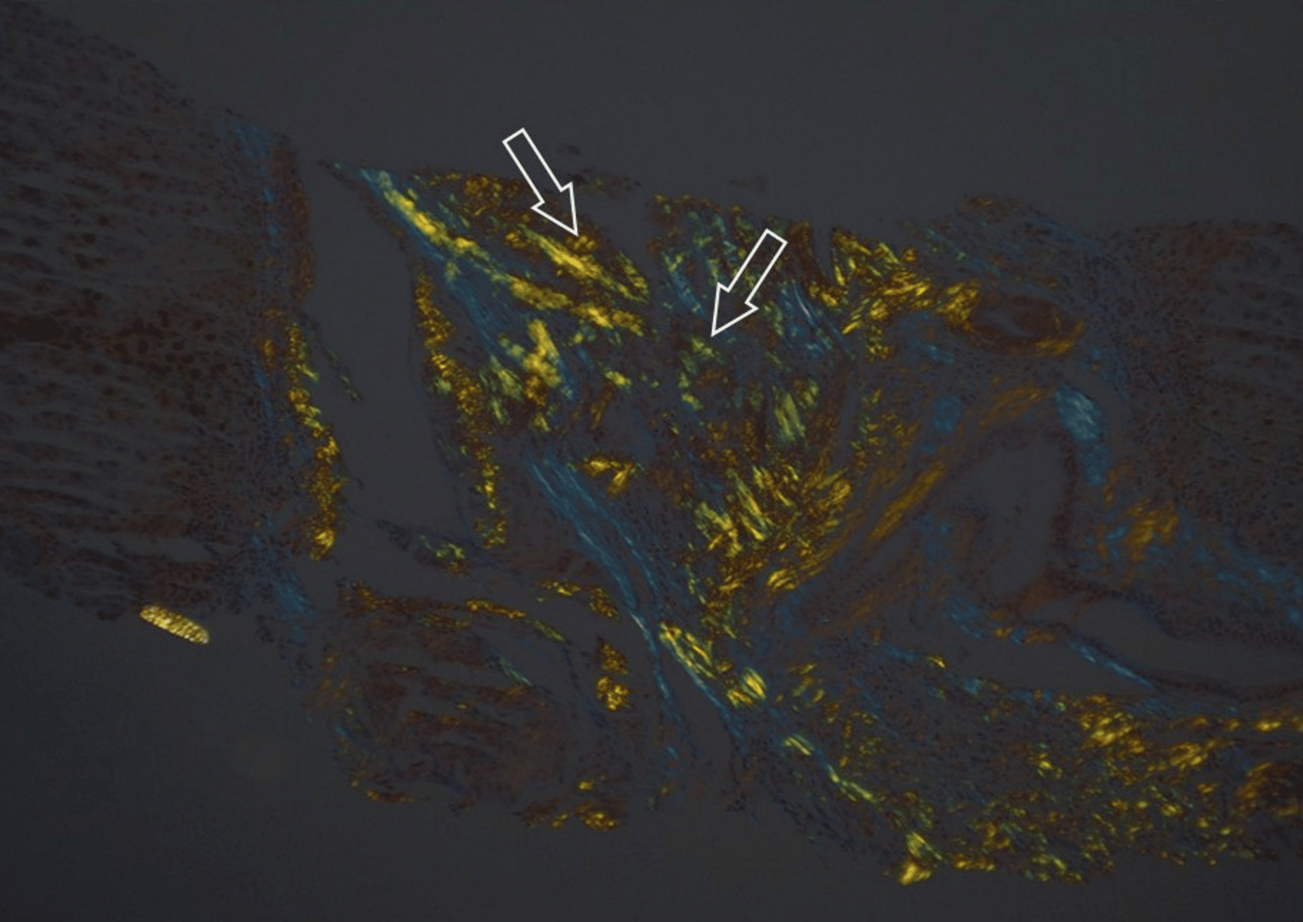
Apple-green birefringence of hepatic amyloid deposits under polarized light Liver biopsy (Congo red, polarized light, ×10) showing apple-green birefringence (arrows), confirming amyloid deposition.

Under polarized light, the Congo red-stained liver biopsy demonstrated apple-green birefringence, confirming amyloid deposition (Figure 2). Liquid chromatography-tandem mass spectrometry (LC-MS/MS) was performed on peptides extracted from Congo red-positive, microdissected areas of the paraffin-embedded specimen, and a peptide profile consistent with AL lambda-type amyloid deposition was detected.

Subsequent hematologic workup identified an associated monoclonal gammopathy. Serum protein electrophoresis demonstrated a restricted band, or M-spike, migrating in the gamma globulin region, and serum immunofixation showed an IgG lambda monoclonal protein. Serum free light chain testing revealed a kappa free light chain level of 14.9 mg/L, a lambda free light chain level of 99.5 mg/L, a kappa/lambda ratio of 0.15, and a difference between involved and uninvolved free light chains (dFLC) of 84.6 mg/L, consistent with lambda light chain predominance. These findings established a diagnosis of systemic AL lambda amyloidosis with hepatic involvement associated with a lambda-restricted plasma cell neoplasm. Factor X activity was reduced at 39% (reference range: 70%-120%), suggesting an acquired coagulation abnormality in the setting of systemic amyloidosis. 

**Figure 3:**
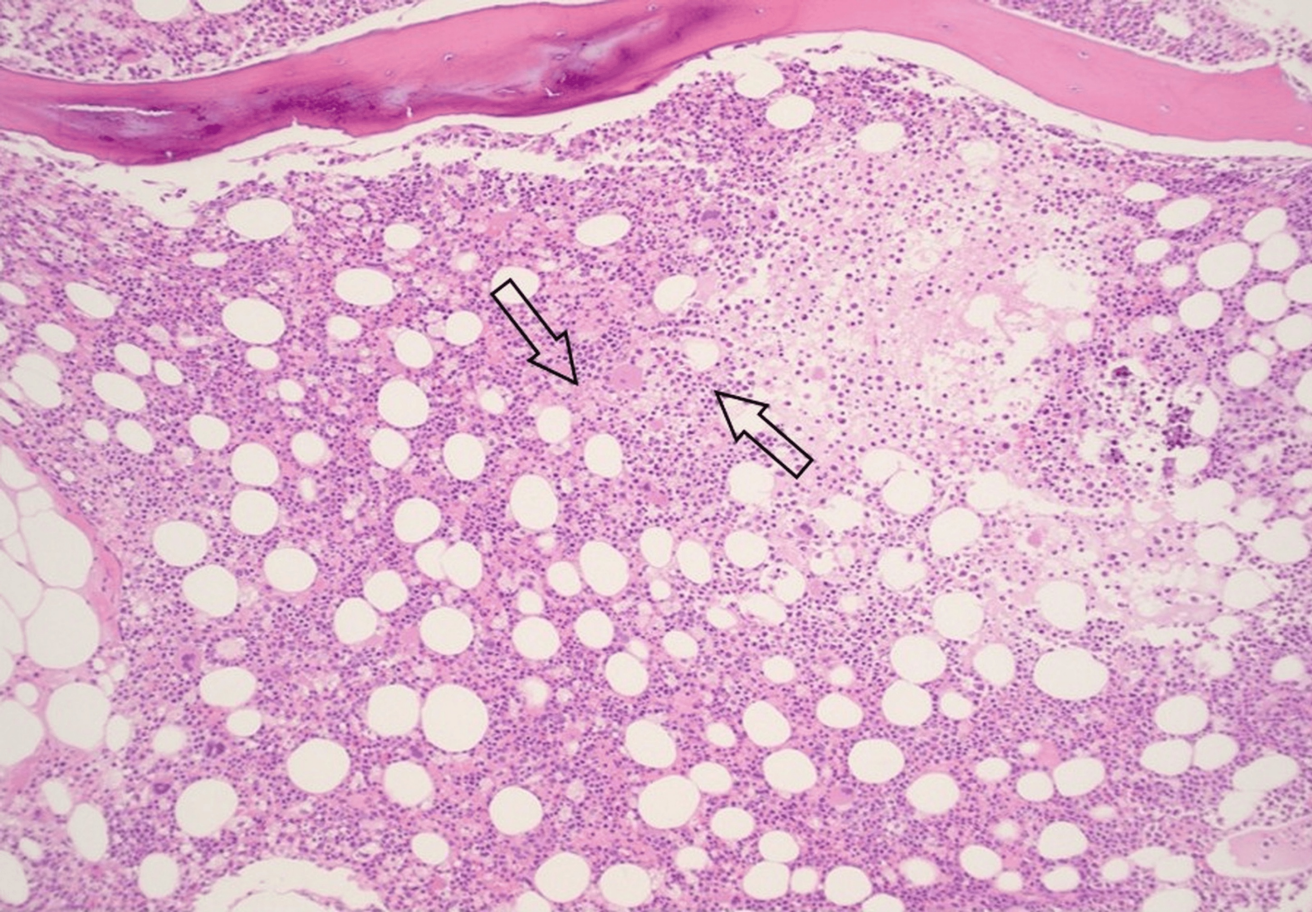
Bone marrow plasma cell infiltration Bone marrow biopsy (H&E, ×10) showing hypercellular marrow with increased plasma cell infiltration (arrows). H&E: hematoxylin and eosin

**Figure 4:**
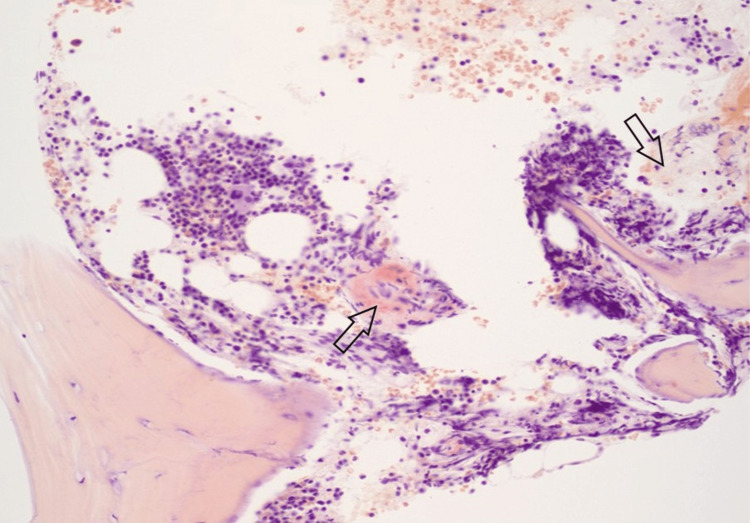
Congo red-positive amyloid deposition in bone marrow Bone marrow biopsy (Congo red, ×10) showing salmon-pink amyloid deposits (arrows) within the marrow.

Bone marrow biopsy revealed a hypercellular marrow, with approximately 70% cellularity, involved by a plasma cell neoplasm comprising approximately 25% of marrow cellularity (Figure 3) and demonstrating lambda light chain restriction. Congo red staining was positive for amyloid deposition, further supporting the diagnosis (Figure 4).

**Figure 5:**
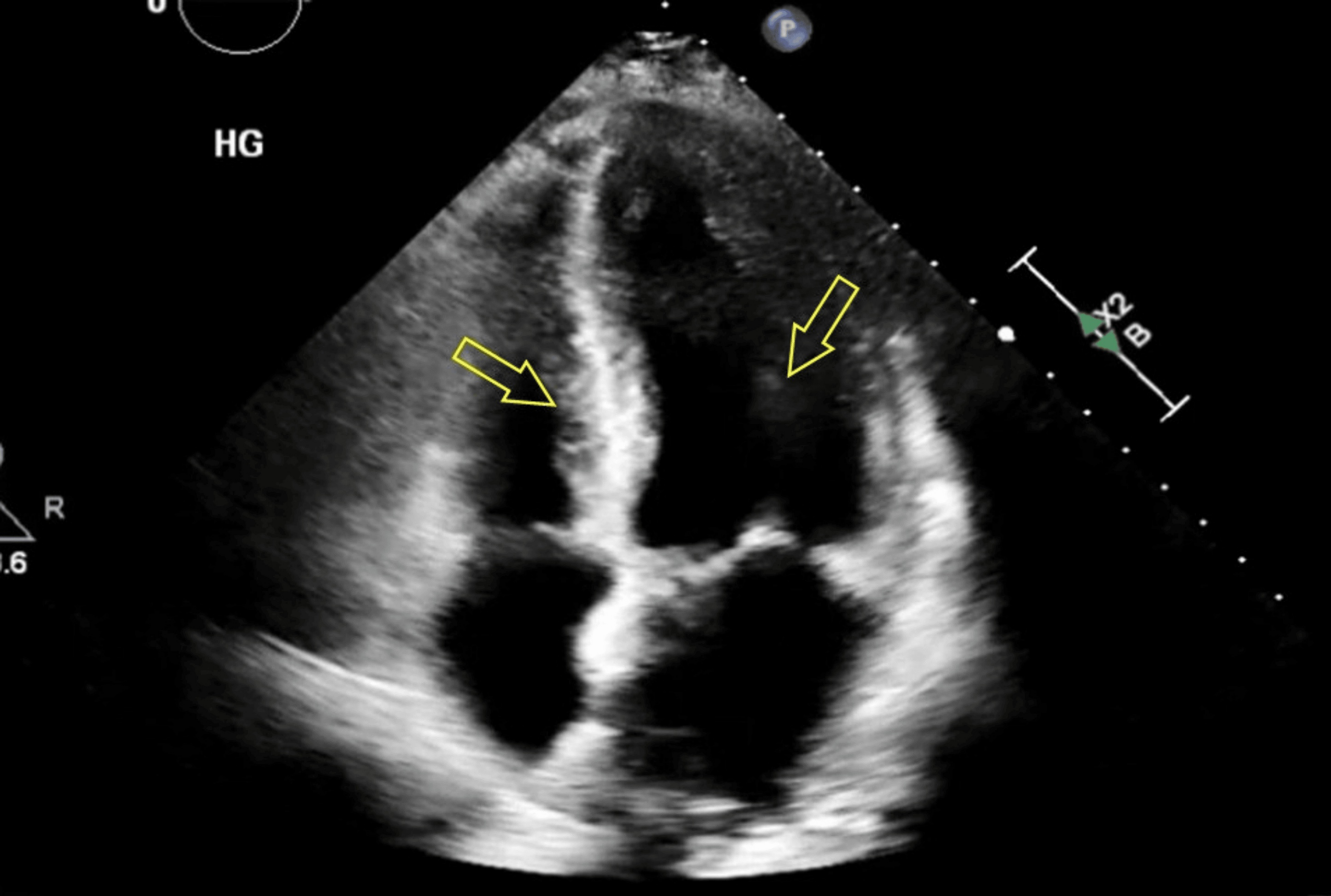
Echocardiographic findings suggestive of cardiac amyloidosis Transthoracic echocardiogram, apical four-chamber view, showing increased left ventricular wall thickness (arrows), suggestive of cardiac amyloidosis.

Although subsequent evaluation revealed multisystem disease, the initial and clinically dominant manifestation was severe cholestatic jaundice caused by hepatic amyloid infiltration, without radiographic evidence of biliary obstruction. Additional clinical findings suggested gastrointestinal and autonomic involvement, including early satiety and dry heaves. Cardiac involvement was suspected based on an elevated N-terminal pro-B-type natriuretic peptide (NT-proBNP) of 2182 pg/mL, elevated cardiac troponin T of 36 ng/L, and echocardiographic findings of severe concentric left ventricular hypertrophy (Figure 5) with preserved ejection fraction and abnormal diastolic parameters. Renal involvement was suggested by proteinuria, with 24-hour urine protein of approximately 1 g and a urine protein-to-creatinine ratio of 965 mg/g, although renal function was initially preserved.

The patient was diagnosed with advanced systemic AL amyloidosis with hepatic-dominant initial presentation and evidence of cardiac, renal, and suspected gastrointestinal/autonomic involvement. She was initiated on attenuated therapy with daratumumab, bortezomib, and dexamethasone. Cyclophosphamide was deferred initially due to significant hepatic dysfunction.

In March 2026, she was admitted for acute decompensated heart failure characterized by worsening lower extremity edema and volume overload. Imaging obtained during that admission incidentally revealed new splenic infarcts. Transthoracic echocardiography findings were highly suggestive of cardiac amyloidosis. Transesophageal echocardiography did not reveal intracardiac thrombus, and further evaluation with cardiac magnetic resonance imaging was planned.

Despite ongoing therapy, her clinical condition continued to deteriorate. In April 2026, she was readmitted with acute kidney injury, worsening cholestatic liver dysfunction, hyponatremia, and acute-on-chronic congestive heart failure. Urine output had progressively declined over the preceding week. Serum creatinine increased to 4.03 mg/dL from 1.27 mg/dL seven days earlier, and potassium rose to 5.3 mmol/L. Total bilirubin increased from 12.6 mg/dL to 17.6 mg/dL within one week, with persistently elevated alkaline phosphatase of 1499 U/L. The patient’s serial laboratory trends and key clinical events are summarized in Table 1, highlighting the rapid progression from hepatic-dominant presentation to multiorgan failure. 

**Table 1:** Serial laboratory trends and key clinical events demonstrating rapid multiorgan deterioration

Time point	Key clinical event	Total bilirubin, mg/dL	Alkaline phosphatase, U/L	Creatinine, mg/dL	Key notes
January 2026 initial admission	Painless progressive jaundice; liver biopsy performed	11.8	>2300	Preserved	No biliary obstruction, ductal dilation, focal hepatic lesion, or hepatomegaly
March 2026 admission	Acute decompensated heart failure and volume overload	12.6	1451	1.27	Splenic infarcts noted; echocardiography suggestive of cardiac amyloidosis
April 2026 admission	Acute kidney injury, worsening cholestasis, and refractory volume overload	17.6	1499	4.03	Progressed to anuric renal failure and transitioned to comfort-focused care

A multidisciplinary team, including nephrology, cardiology, hepatology, and hospital medicine, was involved in her care. She was treated with intravenous furosemide 40 mg every eight hours with strict intake and output monitoring. However, she developed refractory volume overload and progressed to anuric renal failure.

Given rapidly progressive multiorgan failure secondary to advanced systemic AL amyloidosis with severe hepatic, cardiac, and renal involvement, renal replacement therapy was offered. After comprehensive goals-of-care discussions, the patient elected to forgo dialysis and transition to comfort-focused management. She was subsequently discharged to hospice care.

## Discussion

Systemic AL amyloidosis is a rare plasma cell disorder characterized by extracellular deposition of misfolded immunoglobulin light chains, leading to progressive organ dysfunction. The clinical presentation is heterogeneous and depends on the organs involved. Hepatic involvement is well recognized but is often clinically silent or manifests only with hepatomegaly and cholestatic liver enzyme abnormalities. In contrast, cholestatic jaundice as the initial and dominant presenting manifestation is uncommon, particularly in the absence of hepatomegaly or radiographic biliary obstruction.

Our case is notable because the patient presented with painless progressive jaundice and a marked cholestatic pattern of liver injury, with substantially elevated alkaline phosphatase and gamma-glutamyl transferase but only mild transaminase elevation. Initial imaging showed cholelithiasis, but no biliary obstruction or ductal dilation, and serologic evaluation for common autoimmune, viral, and other hepatobiliary causes was unrevealing. Liver biopsy ultimately established the diagnosis by demonstrating amyloid deposition predominantly around the portal veins, along with canalicular and chronic cholestasis, periportal fibrosis, and focal bridging fibrosis. This highlights the importance of considering hepatic amyloidosis in the differential diagnosis of unexplained cholestatic jaundice, even when classic findings such as hepatomegaly are absent.

Marked elevation of serum alkaline phosphatase has been described as an important clue to hepatic involvement in AL amyloidosis. In a large clinical series of primary hepatic AL amyloidosis, unexplained elevation of alkaline phosphatase, proteinuria, and evidence of hyposplenism were noted as diagnostic clues, while poor prognostic factors included congestive heart failure, elevated bilirubin concentration, and thrombocytosis [6]. In our patient, the extremely elevated alkaline phosphatase, progressive hyperbilirubinemia, proteinuria, splenic infarcts, and subsequent decompensated heart failure were consistent with advanced systemic disease and a high-risk clinical course. The marked thrombocytosis at presentation, together with subsequent splenic infarcts, may also suggest functional hyposplenism, a reported diagnostic clue in hepatic AL amyloidosis [6]. 

The prognosis of hepatic AL amyloidosis depends heavily on the degree of liver dysfunction and the extent of extrahepatic organ involvement. Patients with isolated or mild hepatic involvement may have a more indolent course, but those with progressive cholestasis, hyperbilirubinemia, cardiac involvement, renal dysfunction, or gastrointestinal/autonomic manifestations often experience rapid clinical deterioration. Reports of hepatic amyloidosis with severe cholestasis describe poor outcomes, particularly when diagnosis is delayed, or patients are too clinically unstable to receive definitive plasma cell-directed therapy [7]. Our patient developed progressive hepatic dysfunction, decompensated heart failure, splenic infarcts, acute kidney injury, refractory volume overload, and ultimately anuric renal failure within a short interval, reflecting the aggressive nature of advanced multisystem AL amyloidosis. 

Prognostically, this patient had several high-risk features. The revised Mayo 2012 staging system for AL amyloidosis incorporates cardiac troponin T, N-terminal pro-B-type natriuretic peptide (NT-proBNP), and the difference between involved and uninvolved free light chains (dFLC) [8]. One point is assigned for each abnormal biomarker: cardiac troponin T ≥0.025 ng/mL, NT-proBNP ≥1800 pg/mL, and dFLC ≥18 mg/dL, corresponding to stages I through IV and median overall survivals of approximately 94.1, 40.3, 14.0, and 5.8 months, respectively [8].

In our patient, cardiac troponin T was elevated at 36 ng/L, equivalent to 0.036 ng/mL, and NT-proBNP was elevated at 2182 pg/mL. Serum free light chain values were reported in mg/L, with a dFLC of 84.6 mg/L, equivalent to 8.46 mg/dL, which is below the Mayo 2012 cutoff of 18 mg/dL. Therefore, she met two of the three Mayo 2012 biomarker thresholds, most consistent with revised Mayo 2012 stage III disease, which is associated with a median overall survival of approximately 14 months. Her actual clinical course was even more aggressive, likely reflecting the combined burden of severe hepatic, cardiac, renal, and suspected gastrointestinal/autonomic involvement. In addition to cardiac biomarker-based staging, hepatic dysfunction itself carries adverse prognostic significance. Hyperbilirubinemia, particularly total bilirubin greater than 2 mg/dL, has been associated with poor prognosis in hepatic AL amyloidosis; in this patient, the bilirubin was markedly elevated at presentation at 11.8 mg/dL and later increased to 17.6 mg/dL, supporting advanced hepatic involvement and a high-risk clinical trajectory [6].

Prior reports suggest that severe cholestatic jaundice due to hepatic AL amyloidosis is rare but often associated with poor prognosis. Peters et al. reported five cases and reviewed 20 literature cases of primary amyloidosis with severe intrahepatic cholestatic jaundice, noting hepatomegaly in 92%, ascites in 56%, and a median survival of three months after jaundice onset [9]. Yellapu et al. reported four cases of hepatic amyloidosis presenting with severe intrahepatic cholestasis, including two with portal hypertension and esophageal varices [10]. Kim et al. described a similar case of systemic AL amyloidosis with cholestatic jaundice, acute liver failure, portal hypertension, and no hepatomegaly, closely resembling the unusual presentation in our patient [11]. Our case further supports that hepatic AL amyloidosis should be considered in unexplained cholestatic jaundice, even when hepatomegaly and biliary obstruction are absent. 

Early diagnosis is critical because treatment is directed at suppressing the underlying plasma cell clone and reducing production of amyloidogenic light chains before irreversible organ damage occurs. Modern first-line therapy commonly includes daratumumab-based plasma cell-directed regimens. A contemporary review notes that daratumumab combined with cyclophosphamide, bortezomib, and dexamethasone is the only FDA-approved first-line regimen for AL amyloidosis [12]. In the ANDROMEDA trial, daratumumab plus bortezomib, cyclophosphamide, and dexamethasone improved hematologic complete response compared with bortezomib, cyclophosphamide, and dexamethasone alone in newly diagnosed AL amyloidosis [13]. However, treatment may need to be modified in patients with severe hepatic dysfunction or advanced multiorgan failure. In our patient, attenuated therapy with daratumumab, bortezomib, and dexamethasone was initiated, while cyclophosphamide was initially deferred due to significant hepatic dysfunction.

## Conclusions

Severe cholestatic jaundice can be an initial and dominant manifestation of hepatic AL amyloidosis, even in the absence of hepatomegaly or radiographic biliary obstruction. This case emphasizes the importance of considering hepatic amyloidosis in patients with unexplained cholestatic liver injury after common hepatobiliary causes have been excluded. Early liver biopsy and hematologic evaluation are essential for timely diagnosis. However, severe hepatic dysfunction with cardiac, renal, and suspected gastrointestinal/autonomic involvement may indicate advanced systemic disease and is associated with rapid clinical deterioration and poor prognosis despite plasma cell-directed therapy.
